# Sleep architecture and emotional inhibition processing in adolescents hospitalized during a suicidal crisis

**DOI:** 10.3389/fpsyt.2022.920789

**Published:** 2022-08-22

**Authors:** Paniz Tavakoli, Malika Lanthier, Meggan Porteous, Addo Boafo, Joseph De Koninck, Rebecca Robillard

**Affiliations:** ^1^Sleep Research Unit, Institute of Mental Health Research at the Royal, University of Ottawa, Ottawa, ON, Canada; ^2^School of Psychology, University of Ottawa, Ottawa, ON, Canada; ^3^Mental Health Program, Children’s Hospital of Eastern Ontario, Ottawa, ON, Canada

**Keywords:** sleep, inhibition, suicide, emotional processing, adolescence, event related potentials

## Abstract

**Background:**

Suicide is the second leading cause of death in adolescents. Sleep disturbances could alter inhibitory processes and contribute to dangerous behaviors in this critical developmental period. Adolescents in suicidal crisis have been shown to have lighter sleep compared to healthy controls. Additionally, suicidal adolescents have lower neural resources mobilized by emotionally charged inhibition processing. The present exploratory study aimed to determine how sleep architecture in suicidal adolescents may relate to inhibition processing in response to emotional stimuli.

**Methods:**

Ten adolescents between 12 and 17 years of age with a diagnosis of major depressive disorder and who attempted suicide were recruited while hospitalized for a suicidal crisis in a psychiatric inpatient unit. Event-related potentials (ERPs) were recorded prior to bedtime during a Go/NoGo task involving pictures of sad, happy, and neutral faces. Polysomnography was then recorded throughout the night. Pearson correlations were conducted to investigate how inhibition performance and ERP parameters reflecting inhibition processing (i.e., P3d and N2d derived from difference waveform calculated as NoGo minus Go trials) relate to sleep architecture.

**Results:**

Poorer inhibition accuracy in response to emotional stimuli was significantly correlated with shorter REM sleep latency, higher REM sleep, and more frequent nocturnal awakenings. The P3d in response to sad faces was negatively correlated with NREM2 sleep and positively correlated with NREM3 sleep. No such association with the P3d was found for happy or neutral stimuli. There were no significant correlations for the N2d.

**Conclusion:**

Altered sleep in adolescents with depression who are in a suicidal crisisis associated with behavioral inhibition difficulties and fewer neural resources mobilized by inhibitory processes in emotionally charged contexts. This highlights the importance of addressing sleep disturbances while managing suicidal crises in adolescents.

## Introduction

Suicide is the second leading cause of death in adolescents worldwide (1). Adolescence brings physiological, social, and behavioral changes that can increase vulnerability to mental health problems and dangerous behaviors (2). Notably, from childhood to adolescence, changes in sleep co-occur with structural and functional brain changes (3–5), and sleep architecture is sensitive to mental disorders (6–8). Suicidal thoughts and behaviors have been associated with symptoms of sleep disorders among both adults and adolescents (9, 10). In fact, sleep alterations have been identified as a risk factor for suicidal ideations, suicidal behaviors, and death by suicide (11, 12). Although sleep is well known to alter mental health, which can in turn increase suicidality (13, 14), there are some indications that the link between sleep disruptions and suicidality cannot be solely explained by mental disorders (15–18). Notably, This may operate in part *via* sleep-induced alterations in cognitive and emotional processing linked to suicidality (9, 10). For instance, difficulties with inhibitory control, a cognitive function known to be sensitive to sleep loss (19), are thought to contribute to suicidal behaviors (20–23). Inhibitory processes reflect the ability to actively suppress unwanted thoughts or actions (24). This allows individuals to leverage cognitive efforts to suppress habits or impulses and can, therefore, be instrumental for the management of suicidal ideations and behaviors (20, 25, 26). Conversely, weakened inhibitory processes increase impulsivity (27), a phenomenon that can emerge with sleep difficulties (28, 29). From this perspective, the interactions between poor sleep and inhibitory control could possibly increase the risks of engaging in suicidal behaviors. Yet, the contribution of sleep disturbances toward neurocognitive factors underlying suicidality in adolescents remains to be investigated.

### Emotional processing and inhibitory control in the context of suicide

Emotional processing is often altered in suicidal states (30–33). Individuals with suicidal thoughts and behaviors have an attentional bias toward emotionally negative suicide-related cues (30, 33). It has been suggested that vulnerability to suicidal ideations could be worsened by altered inhibitory control, which diminishes one’s ability to inhibit intrusive thoughts (33). Alterations in inhibitory processes can also affect emotional regulation, which may make it more arduous to manage emotionally difficult situations (34–37).

The Go/NoGo task is one of the most commonly used paradigms to study inhibitory processing (38). In this task, participants are instructed to respond when presented with a “Go” stimulus and to withhold their response when presented with a “NoGo” stimulus. The “Go” trials prime the behavioral response while the “NoGo” trials elicit inhibitory processes. Accurate detection of the “Go” stimulus is associated with two main event-related potential (ERP) components [for reviews, see (37, 38)]: (a) the Go-N2, occurring at about 200–300 ms, reflecting the controlled detection of a stimulus event, and (b) the Go-P3, occurring at about 300–500 ms, reflecting decision making and memory updating processes. The “NoGo” stimulus, on the other hand, is associated with (a) the NoGo-N2, reflecting conflict monitoring and detection and, (b) the NoGo-P3, reflecting the actual inhibition process and the conscious decision to withhold a prepared response. These components are thought to reflect the extent of neuronal activity dedicated toward a particular cognitive process.

A larger increase in the amplitude of the N2 from “Go” to “NoGo” conditions has been reported in adults who attempted suicide compared to adults with suicidal ideation (39). Furthermore, we previously observed that adolescents with depression facing a suicidal crisis have a reduced P3d (i.e., difference waveform calculated as NoGo minus Go trials, a marker of inhibition processes isolated from more basic processes) in response to happy and neutral, but not sad stimuli, when compared to healthy controls (40). This suggests that depression/suicidal states may be characterized by difficulties recruiting neural resources to inhibit inadequate responses in certain emotional contexts. We also observed a negative correlation between the severity of suicidal symptoms and the amplitude of the P3d. Furthermore, other studies have shown that self-harming individuals commit more errors in a Stop Signal Task than controls, especially in a negative emotional context (41). Overall, these findings suggest that cognitive resources needed to inhibit negative thoughts in the context of emotional processing can be altered in suicidal individuals (40, 42).

### Sleep, adolescence, and suicidality

Many changes in sleep take place during adolescence, including a reduction in total sleep time, and shortening of the latency to rapid eye movement (REM) sleep (3, 4, 43). The transition into adolescence is also accompanied by an increase in NREM2 sleep (44) with a progressive reduction in slow wave sleep (i.e., NREM3 sleep) and slow-wave activity (SWA, i.e., EEG spectral power in the delta frequency band) toward adult levels (4, 44, 45), and a delay in the sleep wake-cycle (43, 45). This delay in the sleep-wake cycle is often accompanied by circadian disruptions and social jetlag, and these sleep and chronobiologic factors have been shown to alter cognition and mental health (46, 47).

Beyond typical developmental changes, abnormal sleep features have been reported in suicidal adolescents. Notably, Dahl et al. (48) observed that, compared to depressed adolescents and healthy controls, suicidal adolescents exhibited longer sleep onset latency and a marginal increase in REM pressure as reflected by a shorter REM sleep latency with increased time spend in REM sleep and higher REM density. Also, we previously observed that, compared to healthy controls, adolescents in acute suicidal crisis have longer sleep onset latency, higher REM density, and a higher percentage of NREM1 sleep accompanied by a lower percentage of NREM3 sleep in the last third of the night (49). There are also indications that high scores of the suicide item of the Hamilton Depression Rating Scale significantly correlate with a lower percentage of NREM3 in adolescents (50).

To date, little research has been done on the relation between sleep, inhibitory control and suicidality in adolescents. Experimental studies in healthy individuals undergoing partial sleep restriction, a phenomenon akin to naturalistic sleep difficulties, demonstrated that partial sleep loss can lead to heightened impulsivity but would not change the ability to perform while inhibiting responses to negative stimuli on the emotional Go-NoGo task (51). Further work is required to decipher how sleep abnormalities emerging in the context of a suicidal crisis may interact with overt inhibitory control performance and underlying brain processes.

### Objectives

The present report follows our recent findings that suicidal adolescents have altered sleep (49) and a distinct pattern of interactions between emotional and inhibition processing (40). We now aimed to determine how sleep architecture may relate to inhibitory processes in response to stimuli with neutral, positive, and negative emotional valence in these suicidal adolescents. This investigation is exploratory in nature. Nevertheless, based on previous work suggesting that sleep disruptions lead to difficulties in inhibitory control, as well as the typical increase in NREM2 with accompanying reductions in NREM3 occurring both in adolescence and adverse mental health states, it was hypothesized that higher NREM2 and lower NREM3 sleep would correlate with poorer response accuracy on NoGo trials and with a higher amplitude of P3d and N2d evoked by emotional stimuli.

## Materials and methods

### Participants

Participants were sourced from a dataset used in two previous reports (40, 49). The first report compared the sleep architecture of 17 suicidal adolescents to 17 age- and sex-matched controls. The second report assessed the influence of inhibition and emotional valence on behavioral responses and ERPs to the Go/NoGo task across both groups in a subset of participants. From this dataset, all participants with valid sleep and ERP data (*n* = 10) were included in the present report.

All participants were between the ages of 13 and 17 (80% females, mean age = 15.1, *SD* = 1.6 years) and were admitted to the inpatient psychiatric unit of a pediatric hospital due to an acute risk of suicide. The length of stay in this unit typically spans over 5–7 days. Suicidal risk was judged too high for these adolescents to live safely in the community. All participants had a diagnosis of major depression, reported a plan to kill themselves with the full intention of dying, and had made a suicide attempt. Within 24 h of admission, clinical interviews were conducted with the participant and their families by a board-certified psychiatrist to determine diagnosis based on criteria from the Diagnostic and Statistical Manual of Mental Disorders, Fifth Edition (DSM-V; American Psychiatric Association, 2013). Exclusion criteria were: a diagnosis of schizophrenia or neurological or pervasive developmental disorder to limit the effects of sleep and inhibitory process alterations that are known to be associated with these conditions.

Written informed consent was obtained from all participants who were at least 16 years of age. Participant younger than 16 years of age provided assent and their parents provided consent for them to take part in this study. The University of Ottawa’s Health and Sciences Research Ethics Board and the Children’s Hospital of Eastern Ontario Research Institute’s Research Ethics Board approved the study. The study was conducted according to the Declaration of Helsinki.

### Psychological assessment

Depression symptom severity was assessed with the Children’s Depression Inventory Second Edition (CDI-2) (52), which includes 27 items grouped into two major factors, each comprised of two subscales assessing emotional problems (i.e., negative mood/physical symptoms and negative self-esteem) and functional problems (i.e., ineffectiveness and interpersonal problems). Furthermore, the presence and severity of suicidal thoughts and behaviors were assessed using the Suicidal Ideation Questionnaire-JR SIQ-JR; (53) and the Suicidal Behaviors Questionnaire-Revised (SBQ-R) (54). The SIQ-JR includes 15 items assessing thoughts and ideations about suicide. The SBQ-R includes four items addressing different dimensions of suicidality: (1) lifetime suicide ideation and suicide attempts, (2) frequency of suicide ideation over the past 12 months, (3) threat of suicidal behavior, and (4) the likelihood of suicidal behavior. Both the SIQ-JR and the SBQ-R have been found to have good reliability and validity (54, 55).

### Polysomnography

Participants underwent two consecutive overnight polysomnography recordings, according to their habitual sleep-wake schedules. The first night served as an adaptation night while the second night was used for final analyses. Polysomnography was recorded at the patient’s bedside in the inpatient psychiatric unit using the MediPalm 22-Channel polysomnography amplifier (Braebon Medical, Ottawa, ON). EEG data was collected from F3, Fz, F4, C3, Cz, C4, P3, Pz, P4, O1, O2, M1, M2, right, and left electrooculograms (EOG), two chins and two leg electromyograms (EMG), and two electrocardiogram (ECG) channels according to the 10/20 system of electrode placement. A registered sleep technologist manually scored sleep stages according to guidelines established by the American Academy of Sleep Medicine (56) on the Stellate Harmonie (Natus, Pleasanton, CA) analysis software. Total sleep time (TST), sleep onset latency, REM sleep latency, wake after sleep onset (WASO), and absolute and relative sleep stages (NREM1, NREM2, NREM3, REM) were computed. TST was defined as the time spent asleep between the “light off” and “lights on” markers. WASO was calculated as the total duration of all epochs following sleep onset that were scored as wake. Absolute sleep stages were calculated as the total number of minutes participants spent in each respective stage of sleep. Relative sleep stages were calculated as the percentage of time between the “lights off” and “lights on” markers scored as NREM1, NREM2, NREM3, and REM divided by the TST.

### Go/NoGo task

Prior to bedtime on the second night of polysomnography, ERPs were recorded while participants completed an emotional Go/NoGo task. Full details of the task and detailed ERP profiles in the larger sample from which the current cohort is sourced are highlighted in a previous report (40). Briefly, stimuli included images of facial expressions showing happy, sad, and neutral emotions. Five images of males and fives images of females were cropped to an elliptical shape to eliminate hair and background cues. The stimuli were presented in four blocks. Equiprobable neutral and happy faces were presented in random order in two of the blocks. For the first block, participants were asked to press the space bar with their index finger of their dominant hand (Go trials) upon detection of happy faces and to withhold their response (NoGo trials) upon detection of neutral faces. For the following block, participants had to reverse their response procedure by pressing the space bar when seeing neutral faces and withholding their response when seeing happy faces. In the remaining two blocks, the same procedure was done with neutral and sad faces. Each block contained 124 total trials. Each stimulus was presented for 500 ms and the interstimulus interval was 2,000 ms. One block thus lasted approximately 5 min. The total duration of the Go/NoGo task was approximately 20 min. E-Prime software (Psychology Software Tools, Pittsburgh, United States) was used for stimulus presentation and recording of responses.

### Event-related potential recording and analysis

ERPs were recorded during presentation of the Go/NoGo task using BrainAmp amplifiers and Recorder software (Brain Products, Gilching, Germany). The same electrodes used for polysomnography were also used for the ERP recording. The nose served as a reference for all ERP recording channels. Vertical eye movements were recorded from electrodes placed at infra- and supraorbital ridges of the left eye. Horizontal eye movements were recorded from electrodes placed on the outer canthus of each eye. Inter-electrode impedances were kept below 5 kΩ. High-frequency filter was set at 75 Hz. The time constant was set at 2 s. Electrical signals were digitized continuously using 500 Hz sampling rate.

Offline, data were reconstructed using Brain Products’ Analyzer2 Software. The continuous EEG data was bandpass filtered at 0.5–20 Hz (24 dB/octave). A vertical EOG channel was computed by subtracting activity recorded at the supraorbital and the infraorbital ridges of the left eye. The horizontal EOG channel was computed by subtracting activity recorded at the outer canthus of each eye. Independent Component Analysis (57) was used to analyze eye movement and blink artifacts that were statistically independent of the EEG activity. These artifacts were then partialled out of the EEG traces. Continuous data was subsequently reconstructed into discrete single trial 900 ms segments beginning 100 ms before the stimulus onset and then baseline corrected. The segments in which EEG activity exceeded ± 100 μV relative to the baseline was excluded from further analyses. No more than 5% of total trials were rejected from further analyses per participant. Single trials were then sorted and averaged based on stimulus type (Go or NoGo), emotional facial expression (happy, neutral, sad) and electrode site.

### Quantification

The amplitude of all ERP components was quantified for each participant using the means of all the data points within ± 25 ms of the peak amplitude that was identified in the grand average (the average of all participants’ averages). For both Go and NoGo trials, the N2 was identified as the most negative peak between 150 and 300 ms after stimulus onset while the P3 was identified as the most positive peak between 300 and 500 ms. N2 and P3 were identified at the Fz electrode site, where they both tend to be at maximum in amplitude.

Difference waveforms were then calculated by subtracting the ERP waveforms to Go trials from those of NoGo trials to isolate the unique processing following NoGo trials and examine the effects of inhibition (58). From these difference waveforms, the N2d was identified as the most negative-going peak between 150 and 350 ms and the P3d as the most positive-going peak in the window of 300–500 ms, for each emotion. Visual inspection of the ERP waveform revealed that both the N2d and P3d were largest at frontal sites. The data, were, therefore quantified at the Fz electrode site.

### Statistical analyses

Kolmogorov-Smirnov tests confirmed that the data was normally distributed. Pearson correlations were used to assess whether the amplitudes of the N2d and P3d at Fz were associated with the following sleep architecture parameters: sleep onset latency, REM sleep latency, total sleep time, number of nocturnal awakenings, sleep efficiency, and absolute and relative sleep stages (NREM1, NREM2, NREM3, REM). Additional correlations were also conducted to assess potential associations between performance accuracy on NoGo trials (i.e., inhibition condition) and sleep architecture parameters. Subsequently, partial correlations were done for all significant findings to determine whether they survived adjustment for medication intake. Prior to all analyses, outlying values above and below 2SD were curtailed.

## Results

The effects of inhibition and emotional valence on performance indices and ERPs were presented in a previous report based on the larger sample (40). Performance and ERP profiles on the specific subset of participants included in the current report are provided in Supplementary materials.

### Demographics

Participant’s characteristics are listed in Table 1. In brief, depression symptoms severity on the CDI-2 ranged from 11 to 39 (mean = 26.7, *SD* = 10.5) which are within the “high average” or “elevated” severity range. All participants showed clinically significant suicidal thoughts and behaviors based on the standard cut-off score of 8 for the SBQ-R. Suicidal symptoms severity on the SIQ-JR ranged from 24 to 82, with an average score above the standard threshold of 31 (mean = 43.8; *SD* = 17.2), indicating overall significant levels of suicidal ideation.

**TABLE 1:** Demographic and clinical characteristics.

Variables	Participants (*n* = 10)
Age: mean (*SD*)	15.1 (1.6)
Sex distribution [*n* (%) females]	8 (80%)
Medication intake [*n* (%)]	
Antidepressants	9 (90%)
Mood stabilizers/anticonvulsants	1 (10%)
Melatonin	2 (20%)
Stimulants	1 (10%)
Atypical antipsychotics	2 (20%)
CDI-2 [mean (*SD*)]	26.7 (10.5)
SBQ-R [mean (*SD*)]	15.6 (2.5)
SIQ-JR [mean (*SD*)]	43.8 (17.2)

CDI-2, Children’s Depression Inventory Second Edition; SBQ-R, Suicidal Behavior Questionnaire Revised, cutoff > 8; SD, Standard Deviation; SIQ-JR, Suicidal Ideation Questionnaire-Junior, cutoff > 31.

### Correlations between inhibition and sleep parameters

#### Performance accuracy on NoGo trials

**FIGURE 1:**
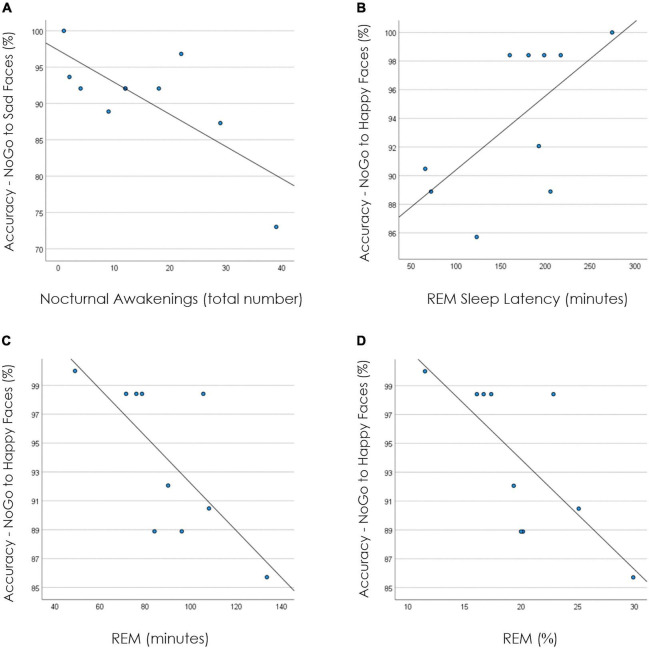
Correlation plots between sleep parameters and performance data. **(A)** Displays the correlation plot for response accuracy on NoGo trials to sad faces and nocturnal awakenings. **(B)** Displays the correlation plot for response accuracy on NoGo trials to happy faces and REM sleep latency (in minutes). **(C)** Displays the correlation plot for response accuracy on NoGo trials to happy faces and REM (in minutes). **(D)** Displays the correlation plot for response accuracy on NoGo trials to happy faces and REM (percentage).

Table 2 shows unadjusted correlation coefficients for the association between inhibition response accuracy (i.e., on NoGo trials) and sleep parameters. Lower response inhibition accuracy on NoGo trials with sad faces significantly correlated with more frequent nocturnal awakenings (*r* = −0.76, *p* = 0.011, Figure 1A). Furthermore, lower response inhibition accuracy on NoGo trials with happy faces significantly correlated with shorter REM sleep latency (*r* = 0.64, *p* = 0.046, Figure 1B) and higher absolute (r = −0.72, *p* = 0.019, Figure 1C) and relative (*r* = −0.74, *p* = 0.014, Figure 1D) amounts of REM sleep.

**TABLE 2:** Correlation coefficients for sleep and response inhibition accuracy.

	Sad		Neutral		Happy	
			
	*r*	*P*	*r*	*P*	*r*	*P*
Sleep latency (min)	0.32	0.368	0.27	0.445	0.14	0.700
REM latency (min)	0.10	0.788	0.00	0.996	0.64*	0.046
Total sleep time (min)	–0.11	0.756	–0.29	0.409	–0.01	0.990
Nocturnal Awakenings (nb)	−0.76*	0.011	–0.62	0.056	–0.56	0.093
Sleep efficiency (%)	0.32	0.361	0.13	0.711	0.26	0.462
Absolute sleep stages (min)						
NREM1	–0.62	0.056	–0.61	0.060	–0.48	0.157
NREM2	–0.32	0.365	–0.24	0.508	–0.30	0.407
NREM3	0.31	0.379	0.34	0.332	0.58	0.081
REM	–0.15	0.673	–0.47	0.176	−0.72*	0.019
Relative sleep stages (%)						
NREM1	–0.60	0.067	–0.59	0.072	–0.49	0.151
NREM2	–0.22	0.551	–0.08	0.829	–0.24	0.507
NREM3	0.33	0.352	0.38	0.283	0.61	0.059
REM	–0.14	0.708	–0.42	0.225	−0.74*	0.014

Inhibition response accuracy as reflected by the percentage of correct responses on NoGo trials in each emotional valence condition.

*Correlation is significant at the 0.05 level (2-tailed).

#### Event-related potentials

##### Conflict detection as reflected by N2d

Table 3 presents unadjusted correlation coefficients linking sleep parameters to N2d amplitudes. There was no significant correlation between sleep parameters and the amplitude of the N2d in response to any type of emotional stimuli (all *p* > 0.050; Table 3).

**TABLE 3:** Correlation coefficients for sleep and conflict detection as reflected by the amplitude of the N2d, at Fz.

	Sad		Neutral		Happy	
			
	*r*	*P*	*r*	*P*	*r*	*P*
Sleep latency (min)	0.51	0.129	0.11	0.761	0.02	0.953
REM latency (min)	–0.35	0.328	–0.05	0.890	–0.10	0.778
Total sleep time (min)	–0.49	0.150	–0.62	0.057	–0.09	0.803
Sleep efficiency (%)	0.23	0.530	–0.32	0.367	–0.06	0.862
Absolute sleep stages (min)						
NREM1	–0.53	0.115	0.20	0.590	0.54	0.106
NREM2	0.02	0.967	0.44	0.200	0.26	0.471
NREM3	–0.10	0.780	–0.54	0.106	–0.36	0.309
REM	0.02	0.952	0.09	0.814	0.18	0.614
Relative sleep stages (%)						
NREM1	–0.48	0.161	0.23	0.523	0.54	0.110
NREM2	0.18	0.619	0.54	0.105	0.22	0.544
NREM3	–0.07	0.847	–0.49	0.155	–0.35	0.329
REM	0.11	0.758	0.18	0.627	0.19	0.599

No correlation reached the significance threshold at the 0.05 level (2-tailed).

##### Inhibition as reflected by the P3d

**FIGURE 2:**
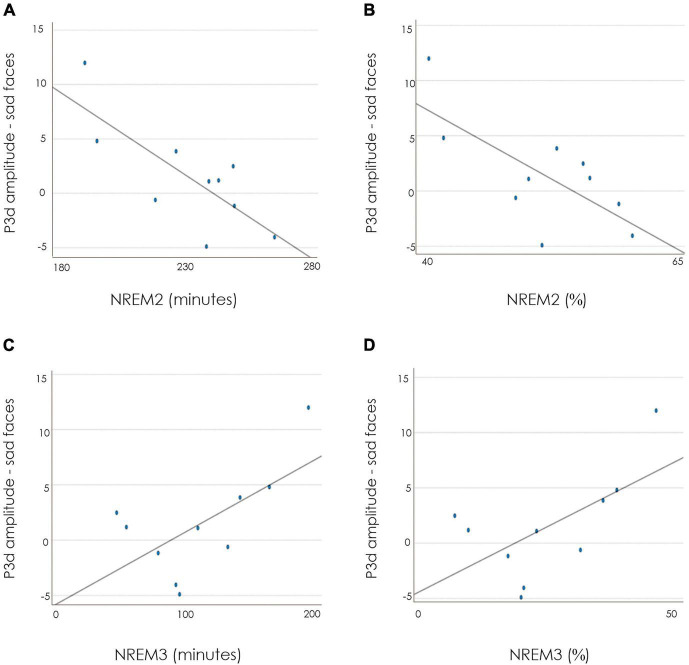
Correlation plots between the amplitude of the P3d to sad faces (at Fz) and NREM2 in minutes **(A)**; NREM in percentage **(B)**; NREM3 in minutes **(C)**; NREM3 in percentage **(D)**.

Table 4 presents unadjusted correlation coefficients linking sleep parameters to P3d amplitudes. The amplitude of the P3d to sad stimuli was significantly negatively correlated with both absolute (*r* = −0.77, *p* = 0.010; Figure 2A) and relative (*r* = −0.71, *p* = 0.022; Figure 2B) amounts of NREM2 sleep. The P3d amplitude to sad stimuli also significantly correlated with higher absolute amounts of NREM3 sleep (*r* = 0.64, *p* = 0.045; Figure 2C), with a similar trend for relative NREM3 sleep (*r* = 0.62, *p* = 0.055; Figure 2D). When controlling for psychotropic medication use, these results remained significant with the exception of the correlation between absolute NREM3 and the P3d to sad stimuli, which became a non-significant trend (*r* = 0.63, *p* = 0.072). Sleep onset latency, REM sleep latency, total sleep time, sleep efficiency, and the time spent in NREM1 and REM sleep were not significantly correlated with the P3d elicited by any type of emotional stimuli.

**TABLE 4:** Correlation coefficients for sleep and inhibition as reflected by the amplitude of Pd3, at Fz.

	Sad		Neutral		Happy	
			
	*r*	*P*	*r*	*P*	*r*	*P*
Sleep latency (min)	–0.03	0.927	0.02	0.954	–0.16	0.658
REM latency (min)	0.09	0.811	–0.59	0.071	–0.29	0.419
Total sleep time (min)	0.33	0.357	0.02	0.950	0.11	0.770
Sleep efficiency (%)	0.29	0.413	–0.03	0.927	–0.15	0.686
Absolute sleep stages (min)						
NREM1	–0.32	0.373	0.05	0.897	0.38	0.285
NREM2	−0.77**	0.010	–0.52	0.120	–0.52	0.123
NREM3	0.64*	0.045	0.27	0.449	0.15	0.676
REM	–0.15	0.684	–0.02	0.952	0.16	0.661
Relative sleep stages (%)						
NREM1	–0.32	0.363	0.07	0.849	0.40	0.258
NREM2	−0.71*	0.022	–0.42	0.233	–0.46	0.179
NREM3	0.62	0.055	0.26	0.471	0.15	0.687
REM	–0.20	0.584	–0.01	0.984	0.15	0.683

**Correlation significant at the 0.01 level (2-tailed).

*Correlation significant at the 0.05 level (2-tailed).

## Discussion

The present study is the first to investigate parallels between brain activity during sleep and brain activity in the wake state during emotionally charged inhibition processing in adolescents undergoing a suicidal crisis. Physiological measures of sleep and inhibitory brain processes were measured in acutely suicidal adolescents while they were hospitalized after attempting suicide, thereby providing information about this critical time window. Findings suggest that lighter sleep, as reflected by higher levels of NREM2 and lower levels of NREM3, is associated with fewer neural resources mobilized by inhibitory processing, as reflected by the amplitude of the P3d in a Go/NoGo task. This was especially prominent in the context of negative emotional valence.

### Task performance and event-related potentials across inhibition and emotional valence conditions

The adolescent brain undergoes significant developmental changes (59) affecting emotional regulation (60–62) and executive functions such as inhibitory control (63–66). As observed in the current subsample, our previous report revealed that adolescents in suicidal crisis take longer time and make more mistakes when processing negative compared to positive emotional stimuli (40), a finding consistent with those reported in another similar study (67). In other words, it may be especially difficult for suicidal adolescents to inhibit inadequate responses when confronted with information with a negative emotional tone, a factor likely to contribute to increased impulsivity (27) in emotionally charged contexts. These cognitive difficulties could notably arise if insufficient neural resources are mobilized to support performance during emotional tasks, a phenomenon that can be appraised using ERPs. As was the case in our previous report based on the larger sample of suicidal adolescents from which the current participants were sourced (40), the subsample of participants included in the current report showed no evidence that conflict monitoring in the early stages of inhibition processing (as reflected by the N2 ERP) varied across emotional conditions. However, the P3 (reflecting later stages of inhibition processing) was significantly larger for negative compared to positive stimuli. This larger P3 could suggest that suicidal adolescents require more neural resources dedicated to withholding a response to negative stimuli compared to positive stimuli. It is important to note that, this larger P3 elicited by negative stimuli compared to neutral or positive stimuli was accompanied by lower inhibition response accuracy. It is thus possible that the extent of neural resources dedicated to the inhibition of negative stimuli was not sufficient to maintain performance levels similar to those observed for positive and neutral stimuli. Overall, the subsample included in the current report had similar performance and ERP profiles than the larger sample of suicidal adolescents included in our previous report (40).

### Association between emotionally charged inhibition and sleep

The novel findings issued from the present report are that inhibition behaviors and underlying neural processes are associated with the extent of sleep abnormalities occurring during a suicidal crisis. From a behavioral perspective, correlations between performance measures and sleep parameters revealed that increased REM pressure and indices of sleep disruptions correlated with poorer inhibition accuracy in the context of emotional processing. More specifically, shorter REM sleep latency and higher amounts of REM sleep were associated with increased failures to inhibit inappropriate response to stimuli with positive emotional valence. This profile of increased REM pressure is a characteristic feature of depression and suicidality in adults [for reviews, see (68, 69)]. Furthermore, higher nocturnal awakenings, a sleep feature previously linked to suicidal states in adolescents (70), were associated with more failures to inhibit inappropriate responses to stimuli with negative emotional valence. Hence, features of sleep alterations commonly emerging in the context of suicidality could possibly influence behavioral inhibition differentially according to the valence of the emotional context. This suggests that poor sleep may be a factor contributing to the difficulties to inhibit suicide-related cues previously reported in suicidal adolescents (30, 33).

These cognitive challenges emerging at the behavioral level co-occurred with signs of altered neural processing. Reduced P3d amplitudes elicited by negative stimuli correlated with higher levels of NREM2 and lower levels of NREM3. A reduced amplitude P3d indicates less differentiation in the amplitudes of the Go- and NoGo-P3 from the raw ERP waveforms and thus suggests that the inhibitory control processes associated with the NoGo-P3 are attenuated. It may be proposed that sleep-related alterations in the recruitment of neural resources for the inhibition of emotionally negative content may diminish the ability to inhibit intrusive suicidal thoughts. More specifically, shallower sleep may be associated with lower resources to inhibit intrusive, negative information.

Although causality cannot be inferred from the present study, these results align with previous experimental sleep deprivation studies demonstrating that sleep plays a casual role in cognitive functions (71, 72) and emotional regulation (73, 74). Notably, research has shown a complex interplay between sleep, emotions and cognition whereby poor sleep leads to increased emotional reactivity and impulsivity, while these daytime experiences can, in turn, affect sleep (75). The current results further reinforce previous knowledge that impaired sleep hampers cognitive inhibitory control (51), a phenomenon also highlighted in the ERP literature. For instance, research has shown a reduction in the amplitude of the P3 component on cognitive control tasks following sleep deprivation (76–78). Furthermore, research examining emotionally charged stimuli has shown enhanced amplitudes of P3/late positive complex (LPP) to negative stimuli following sleep deprivation (79). This suggests greater attention allocation to emotionally negative stimuli following sleep deprivation. It may be postulated that this could be one of the mechanisms *via* which sleep disturbances fuel impulsivity and increase the risk of enacting suicidal behaviors. Increased attention toward negative stimuli may result in an inability to attend to other relevant information. This may be especially problematic for suicidal adolescents who are vulnerable to emotional regulation difficulties. Furthermore, since alterations of inhibitory processes can affect emotional regulation (34), sleep-related alterations in cognitive processing could also indirectly hinder emotional management in the context of a suicidal crisis. Overall, the effects of poor sleep on both inhibitory processes and emotional regulation may heighten the risk for suicidal thoughts and behaviors in adolescents. Further research is warranted to investigate whether reductions in cognitive resources, as a result of disturbed sleep, could affect coping and resilience mechanisms, and ultimately lead to increased difficulties inhibiting suicidal thoughts and behaviors.

The results obtained in this study may also relate to maturational brain changes in the adolescent prefrontal cortex, a brain region highly involved in both inhibitory control (59, 63) and slow wave sleep (i.e., NREM3; 84). Firstly, synaptic pruning in the frontal cortex during adolescence may contribute to the alteration of neural resources available for inhibition processes (80). For instance, inhibition failures in emotional contexts could notably result from a competition between heightened activity in subcortical emotional processing systems and immature top-down prefrontal systems (66, 81). Secondly, the maturational increase in NREM2 sleep (44) and decrease in NREM3 sleep (4, 44, 45) typically observed during adolescence, and commonly associated with adverse mental health states, may contributed to the shallower sleep profile associated with altered inhibition processing. During adolescence, considerable sex differences may influence sleep and inhibition alterations linked to suicidality. For instance, a previous study reported that, compared to their female counterparts, depressed male adolescents had more sleep disturbances characterized by a shorter REM sleep latency, more time spent in NREM1, and less NREM3, especially in the first NREM period (82). In addition, although healthy male adolescents tend to be more impulsive than healthy female adolescents (83), this trend does not seem to apply in depressed adolescents, suggesting that interactions between adverse mental health and sex-related developmental processes may influence impulsivity (84). Also, previous studies reported an association between depression symptoms severity and impulsivity in depressed male adolescents but not in depressed female adolescents (84, 85). The exploration of these sex-based variations may be especially helpful in the context of suicidality in adolescence, a period of sex-specific changes. Although the current study sample had a higher proportion of females and was too small to address this, future studies should assess potential sex differences in the relationship between sleep disturbances and cognitive challenges faced by suicidal adolescents.

Altogether, the combined patterns of performance and brain responses observed in the present study suggest that REM sleep and nocturnal awakenings may be linked to overt inhibition failures during emotional processing, whereas the depth of NREM sleep may be linked to the underlying neural resources involved in emotionally tainted inhibition processing. Overall, suicidal adolescents with lighter sleep may have less neural resources available to inhibit negative intrusive thoughts. This calls for further work assessing whether increased impulsivity following sleep disruptions, particularly in the face of negative emotional information, may increase the risk of enacting suicide behaviors.

### Limitations

This study presents certain limitations. Firstly, the sample size was limited by challenges inherent to this vulnerable population. This small sample size notably prevented the use of multiple regression and sex-based analyses. Nonetheless, previous studies examining the P3 component in depressed and suicidal participants have used similar sample sizes (86–90).

Furthermore, females were overrepresented in this sample (i.e., 80% of all participants). This aligns with expected sex differences in youth hospitalized for suicidal crisis, since it has been estimated that between 10 and 19 years of age, females are 4 times more likely to attempt suicide than males (91). Nevertheless, these sex difference may be influenced by biases inherent to help-seeking behaviors and hospitalizations, and there is a need to further investigate suicidality in adolescent males.

Additionally, the present study cannot dissociate the effects of suicidality from those of depression or other psychiatric comorbidities. Also, this sample was mostly medicated with drugs likely to influence both sleep and ERPs. Nevertheless, depression, comorbidities, and psychotropic medications are highly common in adolescents with suicidal behavior and as such, the results obtained from the present study are likely to be representative of this population.

Considering the high variability in sleep profiles previously observed in youth with mental disorders (6), a single night of polysomnography does not capture potential changes in sleep patterns. Furthermore, the sleep profiles recorded in this study confounds the state of a suicidal crisis with the context of sleeping in a foreign environment. Alterations in sleep patterns could present themselves differently when individuals are sleeping in an inpatient ward compared to when they are sleeping at home. Nonetheless, this study provides a first glimpse of the interaction between cognitive processes and objective sleep metrics within a psychiatric ward, which enables a better understanding of the clinical journey of these individuals.

Although the aim of the present study was to investigate sleep and inhibition control in adolescents with known suicide attempts, future work should assess this association in adolescents who may be at risk, but have not yet attempted suicide. Notably, subsequent longitudinal investigations are required to identify if sleep-related changes in inhibitory control may precede worsening suicidal thoughts and behaviors.

## Conclusion

The present results highlight the relationship between emotionally charged inhibitory control and sleep architecture in adolescents facing a suicidal crisis. This unveils potential mechanisms *via* which poor sleep could possibly worsen neurocognitive processes involved in suicidality. Although they need to be replicated in larger samples, these findings stress the importance of future sleep intervention studies in suicidal youth. Notably, there is a need to assess whether strategies such as adapted pharmacotherapy, psychotherapy, creating a restful environment, limiting daytime naps, and increasing daytime activities could be beneficial to improve sleep, with potential downstream effects on cognitive inhibition that could translate in better management of suicidal ideations and behaviors in hospitalized adolescents.

## Data availability statement

Proposals to access data from this study can be submitted to the corresponding author and may be made available upon data sharing agreement.

## Ethics statement

This study was reviewed and approved by the University of Ottawa’s Health and Sciences Research Ethics Board and the Children’s Hospital of Eastern Ontario Research Institute’s Research Ethics Board. Written informed consent to participate in this study was provided by the participants or their legal guardian/next of kin.

## Author contributions

AB, JD, and RR contributed to the rationale and the design of the study. PT and ML wrote the manuscript. AB carried out the psychiatric assessments. PT and RR contributed to the data collection. PT, ML, RR, and MP conducted analysis of the data. All authors read and approved the final manuscript.
