# Supplementary material for: Sleep architecture and emotional inhibition processing in adolescents hospitalized during a suicidal crisis
Source: Front Psychiatry. 2022 Aug 22;13:920789. doi: 10.3389/fpsyt.2022.920789 (PMC9441873; doi:10.3389/fpsyt.2022.920789)
Supplement: Supplementary file 1 [file Data_Sheet_1.docx]

**[Supplementary Material](https://www.frontiersin.org/about/author-guidelines#SupplementaryMaterial)**

*Tavakoli et al, (2022) Sleep architecture and emotional inhibition processing in adolescents hospitalized during a suicidal crisis. Frontiers in Psychiatry.*

The effects of inhibition and emotional valence on performance indices and event-related potentials (ERPs) issued from the current study were presented in a previous report based on a larger sample which included the current participants (Porteous M, et al, Clinical EEG and Neuroscience. 2021). The sections below provide results from similar analyses on the subset of participants included in the current report. Specifically, a one-way ANOVA with repeated measures on emotion (negative, neutral, positive) was run on reaction times (RT) to Go stimuli. Furthermore, two-way ANOVAs with repeated measures on inhibition (Go and NoGo) and emotion (negative, neutral, sad) were run on accuracy scores, as well as on the raw N2 and P3 amplitudes. Greenhouse-Geiser was used in cases of sphericity violations. Significant main effect and interaction were decomposed with t-tests.

**S1.2 Performance and raw ERP data**

**S.2.1 Reaction times (RT)**

A one-way repeated measures ANOVA was run to compare reaction times to go stimuli across emotional valence conditions: sad (negative), happy (positive), neutral). Overall, RT to Go stimuli varied between 362 and 562 ms. There was a significant effect of emotion condition for RT, F_(1.3, 11.2)_ = 9.2, p = .008, n_P_^2^ = .51 (Figure S1). Specifically, RTs were significantly longer for negative stimuli (mean = 463.2; SD = 18.3) compared to positive stimuli (mean = 427.2; SD = 18.6; p = .008) and shorter for positive stimuli compared to neutral stimuli (mean = 450.8; SD = 19.4; p <.001). There was no significant RT difference between negative and neutral stimuli (p = .225).

**Figure S1.** Mean reaction time (RT) in ‘Go’ condition for each emotional condition.


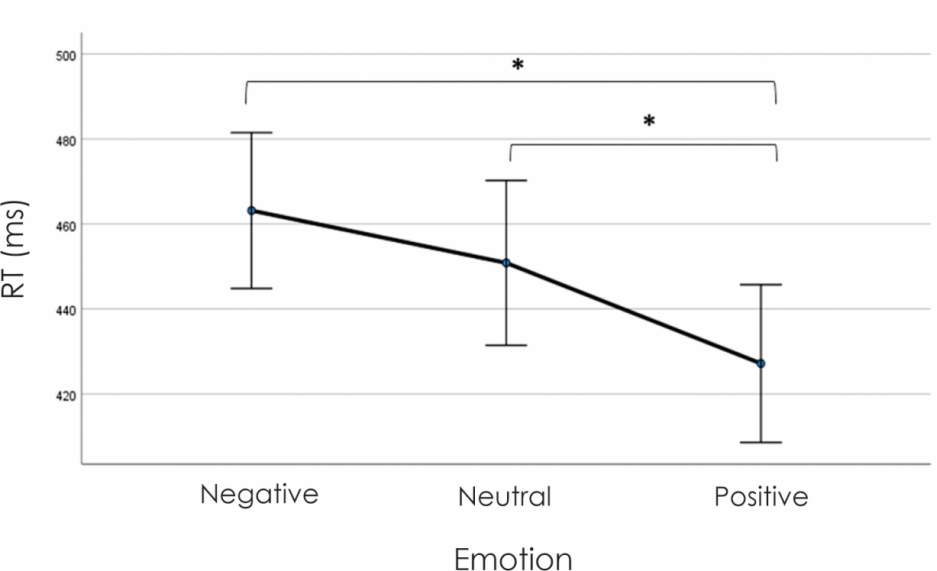


**S.2.2 Accuracy**

Across all inhibition and emotional conditions, 80% of participants had an accuracy of at least 85%. Repeated measures ANOVAs with 2 factors (inhibition; Go vs No Go, and emotional valence: sad (negative), happy (positive), neutral) were run on accuracy. A significant main effect of inhibition, F_(1, 9)_ = 8.7, p = .016, n_P_^2^ = .49 (Figure S2) revealed that the accuracy in NoGo conditions was significantly lower than in Go conditions.

**Figure S2.** Mean accuracy in ‘Go’ and ‘NoGo’ conditions.


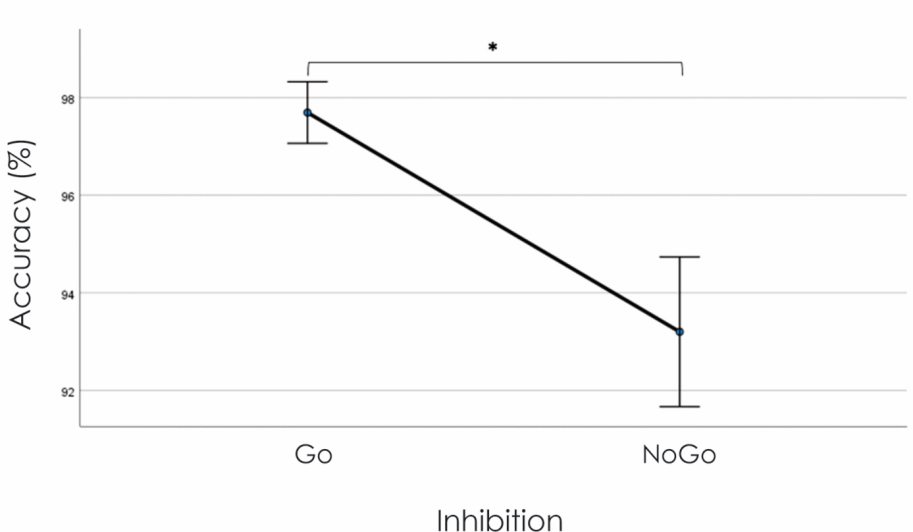


A significant main effect of emotional conditions was found (F_(2, 18)_ = 8.1, p = .003, n_P_^2^ = .47; Figure S3): accuracy was significantly lower for negative stimuli than for both the neutral (p = .022) and the positive (p = .021) stimuli. However, there was no significant difference in accuracy between the positive and neutral stimuli (p = .857). There was also no significant interaction between inhibition and emotion (F_(1.5 13.2)_ = 0.5, p = .551, n_P_^2^ = .06).

**Figure S3.** Mean accuracy for each emotional condition.
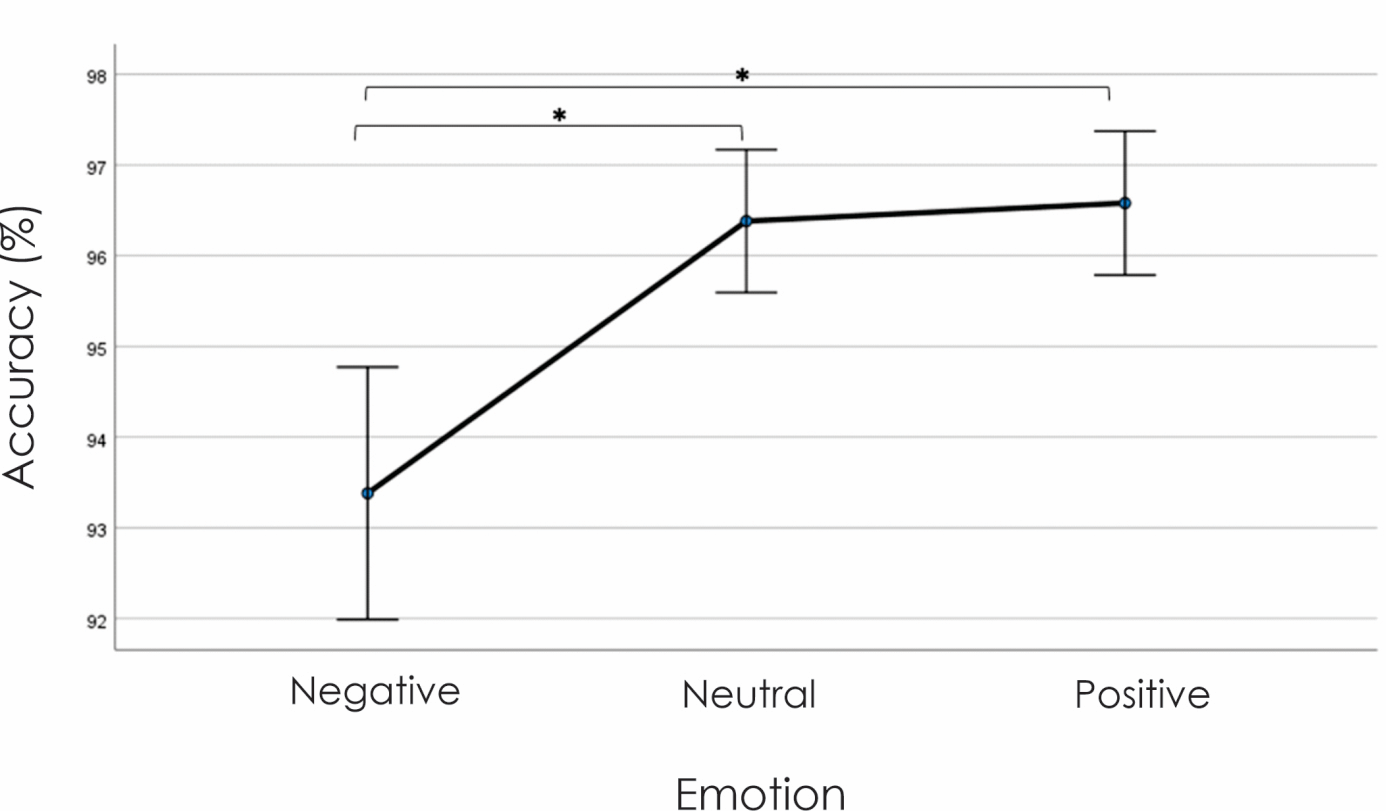


**3.2.3 ERPs**

**RAW ERPs**

Figure S4 presents the Go and NoGo ERP waveforms to the different emotional conditions. Repeated measures ANOVAs with 2 factors (inhibition; Go vs No Go, and emotional valence: sad (negative), happy (positive), neutral) were run on raw ERP components (N2 and P3). A significant main effect of inhibition was found for the N2 amplitude (F_(1, 9)_ = 6.7, p = .030, n_P_^2^ = .43), where the N2 was significantly larger for the Go compared to the NoGo condition.  For the N2, there was no significant main effect of emotion (F_(2, 18)_ = 0.86, p = .440, n_P_^2^ = .09) or significant inhibition by emotion interaction (F_(2, 18)_ = 0.31, p = .737, n_P_^2^ = .03).

A significant main effect of emotion was found for the P3 (F_(2, 18)_ = 3.9, p = .041, n_P_^2^ = .30), where the P3 was significantly larger for negative compared to positive stimuli (p = .048). The P3 elicited by neutral stimuli did not differ significantly from that elicited by negative (p = .091) or positive (p = .186) stimuli. For the P3, there was no significant main effect of inhibition (F_(1, 9)_ = 0.30, p = .597, n_P_^2^ = .03) or inhibition by emotion interaction (F_(2, 18)_ = 0.38, p = .693, n_P_^2^ = .04).

**Figure S4:** Go and NoGo ERP waveforms to the different emotional conditions.

**
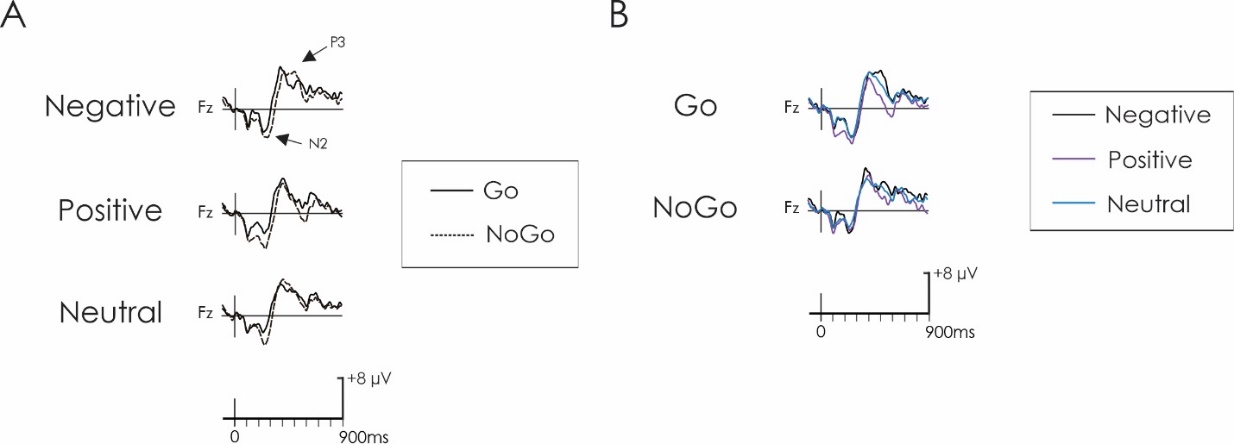
**

**Difference wave components**

Tables S1 provide mean N2d and P3d amplitudes across emotional conditions in the subsample included in the current report. Repeated measures ANOVAs with one factors (inhibition; Go vs No Go) were run on difference waveform ERP components reflecting the change from go to no go conditions. There was no significant effect of emotion conditions for neither the N2d (F_(2, 18)_ = 0.8, p = .466, n_P_^2^ = .08) nor the P3d (F_(2, 18)_ = 1.1, p = .352, n_P_^2^ = .11). This discrepancy with our previous results in the larger sample is likely to result from the smaller sample size in the current report.

**Table S1**. N2d and P3d amplitudes across emotional conditions

|  | N3d | |  | P3d | |
| --- | --- | --- | --- | --- | --- |
|  | Mean | SD |  | Mean | SD |
| Happy (Positive) | -1.84 | 3.16 |  | -0.58 | 3.58 |
| Neutral | -2.86 | 3.13 |  | 0.39 | 2.61 |
| Sad (Negative) | -4.25 | 5.77 |  | 1.47 | 4.84 |

SD: Standard Deviation.
